# Recognition at the Heart of the Complex Situations Experienced by People With Chronic Musculoskeletal Pain

**DOI:** 10.1111/hex.70129

**Published:** 2024-12-29

**Authors:** Jessica Mellier, Aurélie Balis, Fabian Defraine, Quentin Vanderhofstadt, Léa Di Biagi, Marco Schetgen, Pierre D'Ans, Jennifer Foucart, Céline Mahieu, Ana Bengoetxea

**Affiliations:** ^1^ Osteopathy Sciences Research Unit (URSO) Université Libre de Bruxelles (ULB) Brussels Belgium; ^2^ Haute Ecole Libre de Bruxelles HELB Brussels Belgium; ^3^ Department of General Medicine Université Libre de Bruxelles (ULB) Brussels Belgium; ^4^ Research Center in Social Approaches to Health Université Libre de Bruxelles (ULB) Brussels Belgium; ^5^ Research Unit in Motor Psychophysiology Université Libre de Bruxelles (ULB) Brussels Belgium

**Keywords:** adaptation to work, chronic pain, complex situation, health policy, inequality, musculoskeletal pain, recognition

## Abstract

**Objective:**

Chronic musculoskeletal pain (CMSP) is frequent in chronic diseases, decreasing the quality of life of these patients. In a survey conducted in Belgium in 2019, chronic pain was named by patients as the main factor of complexity in their lives. The objective of our research was to provide elements to understand why and how CMSP contributes to the complexity of these people's lives.

**Design:**

Qualitative study through semi‐structured interviews.

**Setting:**

The study was conducted in Belgium with French‐speaking individuals. The interviews took place at the university, in a teaching hospital, in private clinics or in individuals' homes.

**Participants:**

We included 24 individuals with CMSP and living complex situations. The recruitment was made in two phases in agreement with the grounded theory methodology and to reach the saturation of ideas.

**Results:**

The complexity experienced by people with CMSP turns around the notion of recognition, which can be broken down into 3 spheres: intimate, social and legal. The poor quality of listening and the fragmented vision of aid and care professionals generate a feeling of loneliness and incomprehension in the face of illness. The unsuitability of the world of work, the opacity of protocols and the attitude of medical experts reveal inequalities in access to recognition for immigrants and people of low socio‐professional status.

**Conclusion:**

The complexity of the situations experienced could be reduced by implementing health policies that facilitate: legal recognition of this illness; adaptability in the workplace; raising awareness about pain mechanisms; the risks of stigmatization and the need of interprofessional collaboration.

**Patient or Public Contribution:**

Patients and the public participated in the dissemination of our research and were able to help us with recruitment through social networks (call for participation published on Facebook by the association ‘Aidants proches’) or word of mouth. The presentation of preliminary results at conferences as well as the publication of a public article in Belgian journals (http://www.lejournaldumedecin.com/magazine/douleurs-chroniques-un-veritable-parcours-du-combattant/article-normal-63055.html?cookie_check=1671467500%22) facilitated the contact with the public.

## 1 Introduction

### 1.1 Chronic Pain and Bio‐Psycho‐Social Impact

In 2020, the International Association for the Study of Pain (IASP) defines pain as: ‘An unpleasant sensory and emotional experience associated with, or resembling that associated with, actual or potential tissue damage’ [1]. This experience is essentially subjective and influenced by a wide range of contextual factors in the biomedical, emotional, cognitive, spiritual, social and cultural domains [2, 3, 4].

According to IASP and taken up by national guidelines as SIGN [5], pain is chronic if it has been present for more than 12 weeks. It is important to note that 19% of Europeans, including around 24% of Belgians, have chronic pain [6, 7, 8] and that chronic musculoskeletal pain (CMSP) is the most common cause of work‐limiting health problems in developed countries [9].

The presence of chronic pain may induce comorbidities [10, 11, 12] and serious repercussions in all dimensions [13] of the lives of those affected. It increases the risk of developing depression, with impaired sleep [14, 15] and increased psychological stress, which leads to a reduction in daily, professional, social and family activity [7, 9, 14, 16, 17, 18]. People who report CMSP seek primary care for other morbidities more often than people without pain [18]. Over a 10‐year follow‐up period, Lindgren and Bergman found that the presence of CMSP was associated with hospitalizations for conditions such as cerebrovascular disease, ischaemic heart disease, and infections [19]. A systematic literature review on the experience of persons living with chronic nonmalignant pain highlighted an antagonistic struggle in relation to self, time, and others [20]. Individuals must fight to assert themselves, to feel legitimate, to find an explanation for their pain, to apprehend unpredictability, and to negotiate with the health care system.

Indeed, between 40% and 79% of people with CMSP report inadequate pain relief from routine healthcare practices [6, 21]. Long delays are frequent between the onset of pain, first diagnosis, referral and consultation with a specialist [5]. Chronic pain lasts on average of 7.5 years in the Belgian population before one arrives in a pain clinic [6, 22]. In Europe, general practitioners are the first health professionals to be consulted by people with CMSP [6]. This health condition represents more than one‐third of general practice visits [23], indicating a part of primary care workload [24]. All the more, a survey of European general practitioners found that CMSP is one of the most difficult conditions to treat [25, 26, 27, 28], and health systems give it low priority [29, 30].

### 1.2 The Concept of *Complexity* in Health Domain

The notion of *complexity*, as applied to the health domain, appeared in the 2000s, but there is still no definitive definition; probably because the origin and use of the concept have been progressive, drawing on different areas of research not directly related to medicine [31]. It is interesting to note that when seeking to define the concept of complexity in the English‐speaking literature even within the health domain, authors refer to the complexity sciences developed by the Nobel Prize winner Prigogine that involves the study of nonlinear phenomena [31, 32]. French‐speaking literature, however, refers more often to the work of Edgard Morin. For this sociologist and philosopher, complexity etymologically refers to what is ‘woven together’. From this perspective, *complexity* is about understanding the whole, which is more than the sum of the parts, without discrediting the elements that compose the whole. Morin's objective was to change the paradigm of thought so that it was no longer a question of claiming to ‘master reality’, through simplifying thinking, but of practicing thought able to ‘deal with reality’ [33].

In the field of health, complexity is reflected in 3 levels: patient, therapeutic interventions, and public health. The first dimension corresponds to multimorbidity and the sociological vision that includes social and economic status, as well as material living conditions [31]. The second level arises from the complexity of health interventions and can refer to clinical decisions and processes of care that are labelled as ‘non‐standard’, given time commitment and uncertainty involved [34]. Or even, it may refer to a professional's perception of a negative outcome as a result of his or her intervention [35]. The last addresses the complexity of health governance, which brings us back to the term ‘complexity of care’ usually referring to the costs of interventions [36].

In 2019, we conducted a survey in Belgium [37] to identify the factors that are likely to make a health situation complex. The results showed a disparity between heath‐system users' perceptions and those of health professionals. Of 10 factors, chronic pain comes in first position in individuals' responses but is only ranked eighth by healthcare professionals, who considered ‘psychological difficulties’ and ‘difficulty accessing certain care’ as the most important. This finding supports previous studies [35, 38] on primary‐care physicians' perspectives on complex patients, where domains regarding patients' mental illnesses, socioeconomic challenges, and behaviours or traits that complicate chronic disease management were added to the more traditional areas such as the number of illnesses and degree of care coordination [39]. But to our knowledge, there are no studies that specifically explore the notion of complex health situations from the patient's point of view.

Given that patients identified chronic pain as the most important factor making their health situation complex, we wanted to understand why. To do this, we interviewed patients considered ‘complex patients’ by their caregivers and suffering from chronic pain. In this context, the objective of our qualitative research study, focused on patients' experiences, was to provide elements to understand how chronic musculoskeletal pain contributes to the complexity of these people's lives.

## 2 Methodology

### 2.1 Design and Recruitment

The study was conducted in Belgium (Wallonia and Brussels‐Capital). Data collection was performed during October 2019 ‐ March 2020 and February 2021 ‐ February 2022. An interim data analysis was conducted between these time periods.

We used purposive sampling to collect the most diverse set of experiences and insights to achieve external saturation of ideas [40]. The inclusion criteria were adults ( ≥ 18 years) with CMSP, who could understand and express themselves in French, or through family caregiver, who were perceived as a person living a complex situation by health professionals or investigators, and capable of discernment. We based our criteria on the complexity criteria taken from the study by Grant et al. and Loeb et al., which take into account medical (comorbidity, mental health problems, etc.), social (socioeconomic circumstances of the patient) and behavioural factors (personal characteristics of the patient) without forgetting the difficulty of making medical decisions by the professional treating this patient. And we have left the possibility for each health professional to be able, according to their own judgement, to recruit for each of the criteria mentioned. During the first data collection period, we recruited 13 individuals. In alignment with our purposive sampling approach and following multiple rounds of analysis, we adjusted the recruitment criteria for the second data collection phase. The recruitment team was guided to prioritize men, persons residing in Belgium but of non‐Belgian cultural origin, and those from lower socioeconomic backgrounds. During this second period, 12 patients were recruited.

The recruitment was performed by 2 general practitioners, 1 physiotherapist, 4 osteopaths, 1 neurosurgeon, 1 qui qong instructor and 1 receptionist from the ‘Medical Clinic’; through the associations ‘Aidants Proches’ and ‘Infosourds bxl’; and by word of mouth. Contact was made by e‐mail, telephone or directly at the recruiter's premises. Patients had no special relationship with the recruiter and they knew that their care was not in question. Authors were not part of the professionals who recruited, except in the cases where patients contacted them directly after having received information disclosed in meetings, workshops or national journals of disclosure. One interview was excluded because the person no longer met the inclusion criteria at the time of the interview. The sample characteristics are shown in Table 1.

**Table 1:** Demographic Data Table. False names were assigned to the data of each participant. These data were self‐reported, they were not taken from medical records, therefore, the list of comorbidities is not exhaustive. The duration of pain is described in 4 categories: </= 1 year; [1–5 years]; [6–10 years]; >/= 10 years. We made this choice following the results of a retrospective study of 1000 files from a pain clinic in Brussels that indicated that people wait on average 6 years before having access to a specialized interdisciplinary team (unpublished study). The age is indicated by category and the cultural origin of the persons by region to assure anonymity. The socio‐professional categories refer to the INSEE (French National Institute of Statistics and Economic Studies) classification [41]. This category was added as a new criterion of recruitment in the second phase to have more information about the impact of pain in the complex situations lived in the professional sphere.

Subject	Gender	Age	Ethnic origin	Education Level	Socio‐professional category	Pain location	Duration of pain	Work status	Recruited by	Self‐assessment of health	Commorbidities
**Murielle**	W	[30–39 years]	Western Europe	Master	Senior manager and intellectual professions	Thoracic, lower back, pelvis/buttocks	[6–10 years]	Working	Osteopath	Rather bad	/
**Michel**	M	[50–59 years]	Western Europe	Secondary	/	Shoulder, abdomen, lower limbs	[6–10 years]	Disability	Word of mouth	Rather bad	Heart and kidney transplants
**Maxime**	M	[40–49 years]	Western Europe	Master	Senior manager and intellectual professions: consultant	Cervical, lower back, shoulder, abdomen, lower limbs	[1–5 years]	Unemployed	Osteopath	Rather good	childhood cancer, infertility, arachnoidite…
**Catherine**	W	[49–40 years]	Western Europe	Secondary	Intermediary profession: maternal assistant	Cervical, shoulder	[1–5 years]	Sick leave	Osteopath	Rather bad	Depression
**Sophie**	W	[50–59 years]	Western Europe	Bachelier	Intermediary profession: kindergarten teacher	Cervical, shoulder, upper limbs	[1–5 years]	Sick leave	Neurosurgeron	Rather good	Hashimoto's, asthma
**Marianne**	W	[40–49 years]	Western Europe	Bachelier	Intermediary profession: speech therapist	Cervical, thoracic, lower back, shoulder, upper limbs, lower limbs	>/= 10 years	Sick leave	Osteopath	Rather bad	Hypothyroidism
**Yves**	M	[40–49 years]	Western Europe	Secondary	Senior manager and intellectual professions: secondary teacher	Thoracic, abdomen	</= 1 year	Sick leave	Osteopath	Rather bad	Cardia cancer
**Nicolas**	M	[20–29 years]	Western Europe	Bachelier	Employed: sales rep	Lower back, abdomen, lower limbs	</= 1 year	Working	Osteopath	Rather good	Fibromyalgia, repetitive tendinitis
**Simone**	W	[40–49 years]	Western Europe	Bachelier	Intermediary profession: physiotherapist	Upper limbs	[1–5 years]	Working	Neurosurgeron	Very good	/
**Nathalie**	W	[40–49 years]	Western Europe	Secondary	Worker: order picker	Cervical, thoracic, lower back, shoulder, upper limbs, lower limbs, pelvis/buttocks	>/= 10 years	Sick leave	Word of mouth	Rather bad	/
**Sarah**	W	[40–49 years]	Western Europe	Master	Intermediary profession: Midwife	Cervical, thoracic, lower back, shoulder, pelvis/buttocks	[6–10 years]	Sick leave	Word of mouth	Rather good	/
**Emilie**	W	[40–49 years]	Western Europe	Bachelier	Intermediary profession: kindergarten teacher	Cervical, thoracic, lower back, shoulder, upper limbs, lower limbs, pelvis/buttocks	[6–10 years]	Working	General practitionnes	Rather good	/
**Sylvie**	W	[30–39 years]	Western Europe	Master	Senior manager and intellectual professions: marketing, writer	Lower back, abdomen, pelvis/buttocks	>/= 10 years	Transition	Association ‘aidants proches’	/	/
**Henriette**	W	[70–79 years]	Western Europe	Bachelier	Intermediary profession: occupational therapist	lower back, lower limbs	[1–5 years]	Retired	Osteopath	/	/
**Thomas via his mom**	M	[20–29 years]	Western Europe	Secondary	/	cervical, lower back, lower limbs	/	Disability	Association ‘aidants proches’	Rather bad	Deafness, cerebral palsy
**Béatrice**	W	[50–59 years]	Western Europe	Bachelier	Employed: welcomes medical house	Cervical, thoracic, lower back, shoulder, lower limbs, pelvis/buttocks	>/= 10 years	Working	Welcome to the medical house	Rather good	Hypertension
**Mehdi**	M	[40–49 years]	North Africa	Primaire	Worker: construction	Cervical, thoracic, lower back, shoulder, lower limbs, pelvis/buttocks	>/= 10 years	Disability	Physiotherapist	Rather bad	Bowel disorders
**Paola**	W	[30–39 years]	South America	Secondary	Employed: housemaid, hairdresser	Cervical, thoracic, lower back, shoulder, upper limbs, lower limbs, pelvis/buttocks	[1–5 years]	Unemployment	Physiotherapist	Very good	/
**Jasmine**	W	[60–69 years]	North Africa	Primaire	Housewife	Cervical, thoracic, lower back, shoulder, upper limbs, lower limbs, pelvis/buttocks, abdomen	>/= 10 years	Unemployed	General practitionner	Rather bad	High blood pressure, cholesterol, chron disease
**Farida**	W	[30–39 years]	North Africa	Secondary	Housewife	Lower back, upper limbs, lower limbs, pelvis/buttocks	>/= 10 years	Unemployed	General practitionner	Rather good	Depression, addiction
**Camila**	W	[50–59 years]	South America	Bachelier	Employed: multinational	Cervical, thoracic, lower back, shoulder, upper limbs, lower limbs, pelvis/buttocks, abdomen	[1–5 years]	Disability	Physiotherapist	/	/
**Angela**	W	[40–49 years]	Western Europe	Master	Senior manager and intellectual professions: cinema	Lower back, lower limbs, pelvis/buttocks, abdomen	>/= 10 years	Working	Qui Qong classes	Very good	/
**Hamina**	W	[50–59 years]	North Africa	Secondary	Worker: factory	Lower back, lower limbs, pelvis/buttocks, thoracic	>/= 10 years	Disability	Association ‘infosourds’	Rather bad	Thyroid, deafness, diabetes, congenital keratoderma
**Yvonne via her daughter**	W	[60–69 years]	Western Europe	Secondary	Intermediary profession: nursery nurse	Lower back, lower limbs	</= 1 year	Retired	Association ‘aidants proches’	Bad	Breast cancer

The sample included 18 women and 6 men, mean (standard deviation) age 47 (26) years (range 20 to 71 years). Fifteen participants had comorbidities. At the time of the interviews, 8 participants were still employed, 5 were on sick leave, 5 had disability status, 4 were unemployed and 2 were retired.

### 2.2 Data Collection

We conducted individual interviews using a semi‐directive interview grid (Appendix 1). The interviews lasted from 30 min to 1.5 h and were conducted virtually or face‐to‐face (at the university, in a teaching hospital, in private clinics or in individuals' homes). Each interview was recorded with a tape recorder or video‐conferencing system (zoom or jitsi meet) and transcribed manually verbatim, preserving the anonymity of the people and places mentioned. The interviews were conducted by the principal investigator, a clinical osteopath and researcher who was trained in qualitative methodologies as part of his doctoral programme, with the help of two Masters students in osteopathy. This work received approval from the Erasmus Hospital Ethics Committee on October 17, 2019. (Erasmus Reference: P2019/401 and CCB Reference: B406201941028). Each participant gave verbal or written informed consent for participation.

### 2.3 Data Analysis

Atlas.ti software was used for the qualitative data analysis. In accordance with the principles of grounded theory, the data collection phases and the analysis phases took place in an interwoven manner [42]. To refine and complete the analyses, we gradually integrated new cases into the sample, as different as possible from the previous ones. Our analysis therefore gradually stabilized until it reached saturation. The codes were also discussed and defined within an analysis group made up of people from different disciplines (sociology, public health, medical doctor, psychologist, social worker, engineer; all co‐authors), also in compliance with the principles of Grounded theory.

Data collection and coding analysis were carried out in French (native language of researchers who carried out the interviews and coding). The document was written in French and translated to English by a professional translator. The verbatims were translated by a bilingual, native speaker of American English. These latest translations were carried out in close collaboration between researchers and the English translator to accurately capture the intended meaning and emotion behind the colloquial French spoken by the participants.

## 3 Results

### 3.1 Analysis of the Interviews

**Figure 1:**
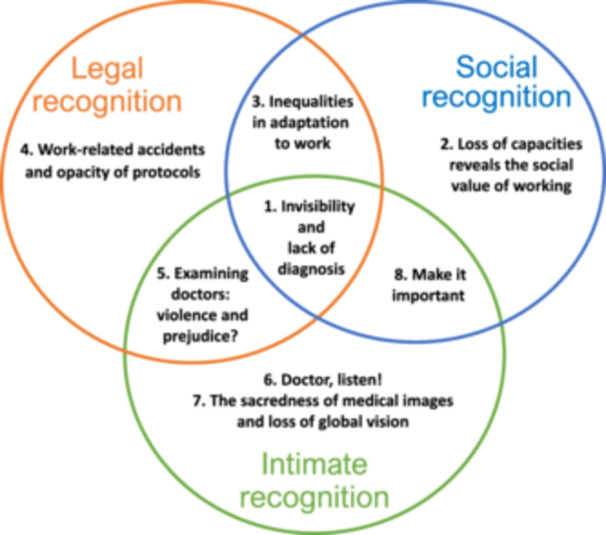
The set of codes was grouped in 3 main categories, which led to the emergence of a conceptual outcome: CMSP disrupts the individual's feeling of recognition. The CMSP‐associated invisibility and the difficulty to have a diagnosis is a central aspect expressed by patients and impacts the 3 levels of recognition. The green circle encompasses all codes linked to intimate recognition. The blue circle brings together all the categories linked to social recognition. The orange circle includes topics related to legal recognition.

In accordance with the principles of Grounded Theory and as indicated above [43], our analysis took place concurrently with the data collection itself, with the categories and concepts emerging gradually and being refined as new interviews were included. The category ‘invisibility and lack of diagnosis’ gradually became the ‘core’ category, enabling the other categories emerging from the data to be organized around it. Grounded Theory recommends using literature to refine emerging categories. We therefore turned to a major author of recognition theory, Axel Honneth. The philosopher and sociologist Axel Honneth distinguishes 3 ‘spheres of recognition’: (1) social recognition linked to skills and work, (2) legal recognition linked to citizen rights and (3) intimate recognition which includes relationships of love and of friendship [39]. Recently, relationship with actors of the care system was added by Koesling and Bozzaro (2021), in their adaptation of Honneth's theory [40]. Our results support their socio‐philosophical analysis [40] and describe how CMSP can impact these 3 recognition dimensions. We have therefore used them to organize our themes. However, our empirical categories are partly located at the intersection between different forms of recognition. This is why we propose to present our results according to the elements included in the following diagram (Figure 1 ).

From our study of factors that make complex the health situation of the 24 interviewed patients suffering from CMSP, 8 categories have emerged: (1) invisibility and the lack of diagnosis, (2) loss of capacities revealing work social value, (3) inequalities in adaptation to work, (4) work‐related accidents and opacity of protocols, (5) examining doctors: violence and prejudice, (6) Doctor, listen! (it's hard to be heard), (7) the sacredness of medical images and loss of global vision and (8) Make it important! This section presents these 8 domains.

### 3.2 Invisibility and Lack of Diagnosis

Pain invisibility and diagnostic difficulties have a jointed impact on the 3 spheres of recognition. Individuals with pain must ‘convince’ both health professionals and those around them of the truth of their complaint. As long as a diagnosis has not been objectively made, doubts may persist within oneself, those around them, among health professionals and decision‐makers.

There was a time when I wasn't sure if it is a pain that we invent ourselves or if it is real. (Guillaume)

CMSP is perceived like ‘is not fatal’; therefore, as a health problem, it may be minimized by caregivers and general population, especially when to compared to cancer.

I have a good friend and colleague who just had breast cancer, at the same time as when I was ill {…} and she said: ‘In fact, everyone is horrified (aghast) for me. Cancer is well known, it's horrible, everyone is there, ‘Are OK? Really OK?’. While you are suffering immensely and nobody acknowledges it.’ (Sarah)

People with CMSP are therefore challenged to show the specificity and severity of their condition in relation to this shared experience. Without imaging or a specific diagnosis, they are sent back to their subjectivity and may not be legally recognized.

*Everyone tells themselves: “All the same. We all have back pain.” From the moment when the rheumatologist started to use the names of the pathologies like ''ankylosing spondylitis, psoriatic arthritis,” they (the insurance representatives) started annoying me less, they were nicer.* (Sarah)

### 3.3 Loss of Capacities Reveals the Social Value of Working

I sometimes get to the point of saying to myself, there's only one thing left for me to do, that's to shoot myself in the head. (…) I am at this point, it's so exhausting. But that, the people around me, they don't understand (…) and I can understand, it's incomprehensible, but I live with it and they don't. So, like: “your pretty uptight again today?”, “OK, shut up, shut up because you are getting on my nerves.” (Nathalie)

When mental health is disrupted, social inclusion can be compromised as participation in social values diminishes. In addition to psychological fragility, the physical decline leading to cessation or loss of employment:

I work in a nursery school. {…} I almost dropped a child and suddenly, I said to myself, ‘I have to stop, it's not possible anymore, it's too dangerous (…).’ (Catherine)

For some patients, nothing is possible anymore, for others who have been able to adapt their work, it becomes the centre of their activities, with nothing being possible alongside it.

I can no longer exercise, do things that I like to do … outside my job.! (Sophie)

Loss of capacity has a direct impact on social recognition. Work appears to be a primordial social value for being an integral part of society. The ‘other’, who has not experienced the persistence of pain in his or her own body, may look critically upon the one who suffers.

I had one of my brothers who said to me: ‘You just need to start working again (…) and you won't be in pain anymore.’ Then, the following year, he slipped and fell on black ice, he broke his foot and now, he has algodystrophy and he is dying from the pain. So, he understood what is pain. He apologized and he said to me: ‘I understand’. (Sarah)

### 3.4 Inequalities in Adaptation to Work

This category emerges through the difference between the stories of people suffering from DCNMS, unlike other aspects developed in our results which emerge from common discourse. The socio‐professional category of the person suffering from CMSP seems to determine the conditions under which the person can or cannot continue to work. Here we have 3 very distinct cases highlighting an inequality of work rights.

Adapted work, that doesn't exist… Just try climbing to the 3rd floor, go down and climb again 10 time per day, you'll see what it's like! (Mehdi)

For Mehdi, a former construction worker of foreign origin, who provided for his family's needs, the work accident was recognized but he feels ‘betrayed’ by the insurance company

I had a 12% disability and they reduced it to 8% because I accepted the “adapted” job.

After 10 years, his health deteriorated significantly following this still very physical work.

He [an expert doctor 10 years later] told me that I was 66% incapacitated for work, because of everything that happened in the meantime.

However, he recognized that ‘it was essential to work’ and that today the most difficult thing ‘is to no longer be able to do anything.’

Camila, an immigrant and administrative employee in a company, was medically recognized as suffering from fibromyalgia; her company refused a reduction in working hours and an adaptation of her workstation.

I worked for 15 years in a company and rose through the ranks…in the professional world, there are very few companies that can accept people with limitations, working hours, modifications to furniture.

For Murielle, of Belgian origin and with an executive/intellectual profession, returning to full‐time work was ‘a victory compared staying stuck at home and should never walk again.’ This was done thanks to the recognition of her employer who was able to adapt the position according to the aims of his employee.

we had new management who was able to recognize my efforts to return to work and who entrusted me with a new responsibility… having changed my work activities and other responsibilities, the job remained fulfilling. It's perhaps that, by the way, the thing that also helped me get back on my feet.

### 3.5 Work‐Related Accidents and Opacity of Protocols

The recognition of a work‐related accident or pathology is not always straightforward. Strict rules, unknown to the workers, appear to exist, for example consulting a doctor on the day of the accident and not returning to work too quickly if the symptoms persist.

my boss told me, ‘sorry, but I didn't report your accident’ and I said ‘why didn't you declare it?’ ‘Ah, because you came back to work 5 days later.’ I said, ‘what the heck?’ If I went to the emergency room, it was for that!’ (…) ‘the insurance company said that I didn't have any rights because I returned to work.’ (Paola)

Individuals may then perceive an injustice that leads them to initiate a dispute with their managers and the medical advisers of insurance companies. Some payers rely on a so‐called procedural flaw to avoid their responsibilities.

The insurance company got involved at the beginning and then said ‘ah, but no, you informed the doctor too late and therefore we will no longer be involved.’ Anyway, there were shenanigans, there were some things that happened that were not clear. Given the fact that I should have declared directly after the accident, something that I had not done (…) the boss categorically refused to recognize his responsibility. (Hamina)

### 3.6 Examining Doctors: Violence and Prejudice?

Consultation with an expert doctor was reported by participants as a mandatory step towards recognizing work‐related accidents or pathologies and/or preventing their recurrence. The cases of Hamina and Paola highlight both the physical and moral violence likely to be present during these consultations, which also determine a crucial part of their future.

Listen, I had just had back surgery. It was barely a month and a half before. I said “wait, I'm not ready to walk yet”. (the physician/specialist) did not take this into account and he all of a sudden lifted my leg, I started crying and said “stop!”. He was not at all apologetic, he was very rigid, very cold. He absolutely did not take my pain into account (…) he closed the file and he sent me his expert assessment, which was… whereas before I had **[a disability]** score of 10 points and normally it should have been considered that way for life, he reduced the score to 7 points. Under normal circumstances, I should have received an increase of my score. (Hamina)

The attitude of the doctors suggests that they suspect the individual of taking advantage of the system.

No, (I don't stay) at home hanging around, watching TV, taking advantage of the fact that I am on sick leave. (Sarah)

The attitudes and relational practices of the medical world have a direct impact also on intimate sphere recognition because they impact the integrity of people with CMSP.

### 3.7 ‘Doctor, Listen!’

Listening is a central element of caregiver–patient relationship, and poor quality of listening seems to be detrimental to people with chronic pain and their pathway through the care system.

the problem is the fact that people, the doctors, don't always understand the kind of pain that one can feel (…) if they can't see something, they don't understand it. Well, I am not going to fraudulently say that I am in pain, to then fake it. If I am in pain, I'm in pain, I'm not trying to make something up, but often, that's the problem with doctors, it's that they don't understand. (Nathalie)

Professionals may doubt the veracity of the person's experience and give insufficient consideration to their complaints, this could lead to poor quality of care, and even to medical errors. The paradox is that some health professionals do not question their own actions and behaviours.

on the other hand, a team (…) that doesn't recognize it's medical errors, of a fractured femur from which everyone can easily recover, I find myself with scars that are due to surgeries that were poorly performed. (…) It's above all the problem, it's the lack of listening, the lack of self‐reflection…. (Muriel)

The interaction between the health professional and the patient is based on a form of hierarchy of knowledge and experience.

They all say that they have access to our files, but no one every consults them. So, as soon as you say something, they look at you in mode ‘wait, are you the doctor?’ so you never dare to contradict and you are not held in high esteem, but you go anyway. (Sylvie)

Lack of listening is present in all subjects, whatever the social class, but the means seem different: for example asking for several opinions, daring to say what you really feel, or disagreeing with something.

### 3.8 The Sacredness of Medical Images and Loss of Global Vision

An opposition between the individual's word and what is experienced as the ‘sacredness’ of images and technical medical examinations on the part of the medical profession, appears in several testimonies.

There is nothing in the ultrasound, then they drag me from department to department, (…) repeat an ultrasound. I had three ultrasounds in all. I had a full scan* (…) then at one moment they say to me ‘there is nothing wrong, that's just that, it's phantom pain, that the way it is. You are going to have to learn to live with it’. (Guillaume)

According to individuals with CMSP, the frequent use of these diagnostic tools is a symptom of this holding sacred the images, which is itself the result of a fragmented vision of the body and of being.

I find that the doctors are too … focused on one part of the body already! (…) sometimes I don't feel like saying much (to the doctor) because I don't want to undergo more (medical) examinations! (…) I feel that I have undergone enough medical exams in my live. That's enough!……… they have a tendency to concentrate too much on one part of the body first, because they don't realize that we are whole (laughter) no, really, it's true. And if they don't know, it's quickly ‘oh, we're going to do a test.’ (Beatrice)

Overuse of (medical) tests hides an ignorance within the medical corps with respect to the global functioning of the body and pain mechanisms. This fragmented vision of one's being:

gives the impression that one is not in good hands. (Sophie)

A second factor that may explain the redundancy of medical images is the poor quality of interprofessional collaboration.

I find that it's deplorable, what is required: if one has a scan somewhere, if you go to another clinic, they do the same again because, I don't know, they don't trust [the first] … or I don't know what … It's not normal! (Maxime)

We observe a lack of collaboration between institutions but also between different health professionals.

I was in despair by the lack of globality. (…) I find it to be very hard because 1 day you go to the ‘naturopath’ [alternative medicine practitioner] who tells you one thing, another tells you something else, you go to the physician at the teaching hospital and he dismisses all the others, saying ‘practitioners of alternative medicine, it's crap’ and in the end … the only thing is that in the meantime it is the natural healers who have helped me the most (…) personally, I find the hardest to swallow is this non‐global approach and the inability to see further than each discipline you see. (Sylvie)

### 3.9 Make It Important

Participants questioned how they should communicate to have hope of being listened to and to be considered. ‘Make it evident’ then appears as a strategy:

One thing that I said to my mom, it's that we are going to stop not complaining. It's paradoxical, I was raised that way, you have pain somewhere, well, keep quiet, it going to go away. Well, no, when one goes to the hospital, you need to complain, because otherwise they don't listen to you. (Yvonne's daughter)

However, this strategy may be detrimental to social and family relationships:

when I say it hurts: ‘Oh, it's OK, it's good, stop complaining.’ so I don't say anything more, I shut my mouth. (Nathalie)

## 4 Discussion

We set out to investigate what makes the situation of chronic pain patients complex, based on the notion of ‘complexity’ currently being used in healthcare research, which defines 3 levels: complexity of the individual cases, complexity of care and interventions, and complexity of needs [31, 34, 35, 36]. Our surprise was to highlight, thanks to Honneth's theory [44] and the adaptation made by Koesling and Bozzaro [45], that, from the patient's point of view, what makes their situation complex is not only focused on pathology or interventions but rather on the lack of recognition that they experience at 3 levels of their life: intimate sphere, social and legal.

Understanding the stories of our subjects through the theoretical framework of *recognition* allows us to identify new dimensions of complexity in the field of health. From the 8 dimensions, we have identified 5 that have already been described in the literature concerning chronic pain viewed from the patients perspective and 3 mark the innovation brought by our study: ‘opacity of protocols for work‐related accidents’, ‘inequalities in adaptation to work’ and ‘Examining doctors: violence and prejudice?’. The first one is related to legal recognition, the second one is in the intersection between legal and social recognition, and the last one is in the intersection between legal and intimate sphere recognition.

### 4.1 Legal Recognition: Reduce Inequalities?

Legal recognition, also known as political or civic recognition, is a historically derived product ‘guaranteeing a person's freedom and equal status’ [45]. Koesling and Bozzaro state that ‘having legal rights is conditional, so controversies arise over the central question, who gets them.’ Our results reveal an inequality of access to this recognition, as it was only during interviews with a population of workers of low socio‐professional status and education level, non‐Belgian cultural origin (albeit officially resident in Belgium) that this reality emerged. Of the two women in our sample who experienced workplace accidents, one was working in industry and the other was working as a cleaner. But the problem is potentially much broader. European statistics for 2021 reveal that nonfatal accidents at work are most frequent in manufacturing (19.2% of the EU total in 2021), human health and social work (13.5%), construction (12.9%) and trade (12.5%) [46]. In the specific cases of work accidents, lack of knowledge of the rules and procedures on the part of workers leads to the refusal of legal recognition of the after‐effects from accidents. Indeed, if a person does not consult a doctor on the same day as the accident or returns to work too quickly, this recognition may be refused. Especially since social security disability insurance requires proof of an observable lesion correlated with the pain, which is currently difficult to objectively quantify [47]. The consequence for individuals is precariousness and unequal access to assistance and care, which may lead to a ‘vicious downward spiral of pain, economic loss, and distress’.

### 4.2 Social‐Legal Recognition: Revolutionize the World of Work?

According to Koesling and Bozzaro, social recognition is based on specific values and ‘when a person matches and satisfies these specific values and thereby contributes to common goals, they are recognized as a valued member in the shared social praxis and can establish self‐esteem’ [45]. However, if for some reason, such as the presence of chronic pain, the individual's contribution and participation in society is restricted, then he or she may be deprived of ‘any opportunity to attribute social value to [his or her] own abilities’. Our results support this point of view and, in the specific case of Belgium, they show the important place that work occupies in social recognition. For the founders of the sociology of work, Friedmann and Naville (1961), ‘Man is a social animal (…) essentially occupied with work.’ The study of an individual's identity has long ago put work at the centre, the other spheres of life being analysed from this ‘fundamental matrix’: family, leisure, religion [48]. However, a French survey of 8403 individuals reveals two constituent elements of what could be called a strong “work identity”: belonging to the social and economic categories and not having a family ‘burden’ [49]. The same study reveals that immigrants attach much greater importance to work than French people born of French parents or people of immigrant descent, even if it is not the main factor determining their identity.

Our study shows that the possibility of continuing to work depends on the possibilities of adapting one's work environment, which seems unequal according to company policies, geographical and cultural background and lower or intermediary socio‐professional status; the latter two being risk factors associated with chronic pain [50]. Being an immigrant is also regarded as a risk factor for the progression of chronic widespread pain into a state of disability [51]. This population is more likely to fight to continue working to be a valued citizen [52]. A study carried out in Sweden on 2268 disabled people revealed that adjusting work schedules and reorganizing tasks were the most important aspects for people with physical disabilities, compared to nonphysical disability for which the need for aids and adaptations differs [53].

This reality gives rise to complex life situations due to its effects. Indeed, social exclusion generates psychological pain that can lead the individual to commit suicide [54], this psychological pain being ‘the emotional result of the frustration of fundamental psychological needs such as self‐esteem, belonging or control’. Thus, being recognized as suffering depends mainly on laws and medical vision [55]. Without such recognition, individuals are identified ‘negatively’ as ‘lazy’ or ‘taking advantage of the medical or social security system’ [45]. Toye et al. refer to ‘loss of personal credibility’ as a consequence in part to stigmatization by employers, leading to diminished self‐esteem [56, 57].

### 4.3 Intimate‐Legal Recognition: Therapist's Attitude

Following on from Koesling and Bozzaro's work, we explore the notion of intimate sphere recognition through the relationship between healthcare professionals and people with CMSP. Invisibility, inherent to chronic pain, would be at the origin of scepticism, suspicion, stigmatization or controversy of this disease because of the impossibility to objectively identify it with medical imagery [57, 58], leading to loss of confidence in oneself and loved ones. The sacredness of these medical images for health professionals takes precedence over listening to the patient, reducing the quality of care, which can even lead to medical errors. This emerging theory is supported by Potier [59], who describes the current existence of a ‘scoping imperative’ which makes vision the guarantee of reliability in the diagnostic process. He even goes so far as to speak of a ‘quasi‐religious effect’, the whole society being ‘amazed, fascinated by a medical progress which it perceives as limitless’. Moreover, the development of these technosciences would ratify, according to him, ‘the representation of a fragmented body, each piece of the body having its own privileged mode of investigation on the technical level’. All of this also contributes to perpetuating dualistic thinking; separating body and mind, and ‘real’ pain from ‘imaginary’ pain [60].

The lack of empathy from some health professionals, also called ‘negative empathy’ [55], and examining doctors' poor bedside manner encourages stigmatization of patients with chronic pain, with stereotypes influencing clinicians’ decision‐making and health policies. A unilateral, imposed and ostensibly ‘objective’ interpretation is at the heart of this stigmatization, with professionals questioning the legitimacy of reasons for complaining or the reality of their symptoms [61]. The question that arises from our results is: would there then be a shift, in the eyes of ‘others’, between the invisibility inherent in suffering and the invisibility of the individual themself through the absence of recognition of their suffering?

## 5 Clinical and Health Policy Implications

To reduce the inequalities between the lives of people suffering from CPMS and the phenomena of precariousness and social exclusion, it seems essential to develop policies that facilitate access to genuinely adapted work, by encouraging employers and companies to adapt working hours and tasks [53]. A second area for improvement would be to integrate the patient into the decision‐making process concerning orientation towards adapted employment, in particular by taking into account his or her skills, desires and life goals [62]. To our knowledge, there are no studies on patient participation in the choice of work adaptation. However, recent studies reveal that even in major healthcare decisions, shared decision‐making is still under‐applied [63].

Political speeches, pain‐related policies, and regulations are also crucial: Pain is deeply political because it is at the heart of debates for framing notions such as suffering, morality, and social well‐being in the context of health insurance or disability benefits [47]. Furthermore, in the world of work, it seems important to improve information regarding the rules for workplace accidents. In many cases, the lack of legal recognition cannot be achieved because the protocols were not followed by the worker, but these workers are not aware of the protocols or the consequences of noncompliance. Our proposal would be that in the same way that risk prevention protocols [64] (e.g. use of helmets or glasses, etc.) have been included in good business practices, the protocols to be followed in the event of workplace accidents should also be explained to workers and they should be helped to follow them.

The exact status of doctors involved in legal recognition is not clear from participants’ testimonials, as there may be confusion between examining doctors for social services and insurers, and occupational physicians. A study carried out in Belgium shows that relations between these professionals can be dysfunctional, even conflictual [65]. Here, we have chosen to use the term ‘medical experts’, which encompasses these different professions.

The development of interprofessional collaboration, including family caregivers and patients, for choosing aids and care, would facilitate the development of a global vision, supporting recognition and innovation in terms of support [66, 67].

## 6 Strengths and Limitations

Flemish‐speaking Belgians were not included in the study because the investigators did not speak the language, and the experience of this population may differ because of the different health policies in the Flemish region. However, the 24 interviews allowed us to reach an external saturation of ideas for Belgian residents who understood and spoke French. Our study thus makes it possible to support and complete, with qualitative data, the adaptation of the theory of recognition to the population with chronic pain [45]. One aspect that emerged despite the fact that no specific questions on this subject were included in the interview grid is the power of recognition that insurers/payers and employers have over their insured or employees. This aspect was highlighted in the last interviews with immigrants and people of lower socioeconomic status. It is this unequal access to recognition, particularly legal recognition, that makes our work original, and our contribution to the definition of complexity in healthcare.

## 7 Conclusion

This study highlights the fact that the lack of recognition is at the heart of the complex situations experienced by people with CMSP. In general, they all feel the need for a change in the relationship with health and support professionals, advocating the necessity for more listening and global vision of being and care. Indeed, inequalities of treatment are apparent for immigrants and people of average or low socio‐professional status mainly for aspects involving legal recognition. The complexity of the situations experienced could be reduced by implementing health policies that facilitate legal recognition of this illness; adaptability in the workplace; raising awareness about pain mechanisms, about risks of stigmatization and about the need for interprofessional collaboration.

## Author Contributions

**Jessica Mellier:** conceptualization, methodology, data curation, formal analysis, visualization, writing–original draft, writing–review and editing, investigation. **Aurélie Balis:** data curation, formal analysis, investigation. **Fabian Defraine:** supervision, formal analysis, methodology. **Quentin Vanderhofstadt:** methodology, formal analysis, supervision. **Léa Di Biagi:** formal analysis, methodology, supervision. **Marco Schetgen:** supervision. **Pierre D'Ans:** project administration, validation; supervision, formal analysis, methodology. **Jennifer Foucart:** formal analysis, methodology, supervision, validation. **Céline Mahieu:** methodology, formal analysis, supervision, validation, project administration, writing–review and editing. **Ana Bengoetxea:** writing–review and editing, validation, funding acquisition, supervision, conceptualization, visualization, formal analysis, project administration.

## Conflicts of Interest

The authors declare no conflicts of interest.

## Supporting information

Supporting information.

## Data Availability

The data that support the findings of this study are available on request from the corresponding author. The data are not publicly available due to privacy or ethical restrictions.
