# Erratum to “Recognition at the Heart of the Complex Situations Experienced by People With Chronic Musculoskeletal Pain”

**DOI:** 10.1111/hex.70184

**Published:** 2025-02-12

M. Jessica, B. Aurélie, D. Fabian, et al., “Recognition at the Heart of the Complex Situations Experienced by People With Chronic Musculoskeletal Pain,” *Health Expectations* 28, no. 1 (2025), https://doi.org/10.1111/hex.70129.

In the published article, there is an inversion between first and last names for all authors.

The correct authors' names read as per below.

Jessica Mellier, Aurélie Balis, Fabian Defraine, Quentin Vanderhofstadt, Léa Di Biagi, Marco Schetgen, Pierre D'Ans, Jennifer Foucart, Céline Mahieu, Ana Bengoetxea

We apologize for this error.

