# Supplementary material for: Recognition at the Heart of the Complex Situations Experienced by People With Chronic Musculoskeletal Pain
Source: Health Expect. 2024 Dec 29;28(1):e70129. doi: 10.1111/hex.70129 (PMC11683188; doi:10.1111/hex.70129)
Supplement: Supplementary file 1 — Supporting information. [file HEX-28-e70129-s001.pdf]

Appendix 1 :

PATIENT INTERVIEW GUIDE:

Starting question: Can you describe your current situation? How do you feel now?	
More specific opening question: Can you tell me about the place of physical pain in your life?	
Complexity:	What do you find complex in your life today?<br><br>What do you find most difficult to live with today? (towards the end of the interview)
History and characteristics of pain:	Can you describe the context in which your pain appeared?<br><br>How do they present themselves today?
Quality of life:	What is the impact of your pain on your daily life?<br><br>Can you tell me about the professional, family and social network around you?<br><br>How do you feel morally?
Care pathway	Can you describe your journey through the health care system?<br><br>What has helped you or could help you?<br><br>Have you been led to consult complementary or alternative therapists? How did this come about in your journey?<br><br>How would you define the quality of care you received?<br><br>If you had a magic wand, what would you improve about the health care system?<br><br>What role do you have in making decisions about your care?<br><br>Today, what are your needs in relation to the support of these pains?<br><br>What do you think is important to improve, to develop, in your life to improve your health?
Interdisciplinary collaboration	Can you tell us about the collaboration between the different professionals you met?
Financial situation	Can you tell us about the impact of pain on your financial situation?
